# Neuroprotective Potential of Melatonin: Evaluating Therapeutic Efficacy in Alzheimer's and Parkinson's Diseases

**DOI:** 10.7759/cureus.50948

**Published:** 2023-12-22

**Authors:** Norris C Talbot, Patrick M Luther, Noah J Spillers, Amanda R Ragland, Evan J Kidder, Rucha A Kelkar, Giustino Varrassi, Shahab Ahmadzadeh, Sahar Shekoohi, Alan D Kaye

**Affiliations:** 1 School of Medicine, Louisiana State University Health Sciences Center, Shreveport, USA; 2 School of Medicine, Medical University of South Carolina, Charleston, USA; 3 Department of Pain Medicine, Paolo Procacci Foundation, Rome, ITA; 4 Department of Anesthesiology, Louisiana State University Health Sciences Center, Shreveport, USA

**Keywords:** alzheimer's disease, parkinson's disease, treatment, sleep disorders, melatonin

## Abstract

Decreased melatonin levels have been linked to both Alzheimer's disease (AD) and Parkinson's disease (PD), which are the two most prevalent neurodegenerative disorders. The development of sleep disorders is widespread in patients diagnosed with AD or PD. In this regard, calcification of the pineal gland, typically seen in the third decade, has been associated with a reduction in melatonin production. Recent studies have suggested that exogenous melatonin application can be utilized to treat sleep disorders in patients with neurodegenerative diseases. Furthermore, research has shown that deficiencies in melatonin levels in patients with AD or PD begin before a diagnosis of either disease is made. These findings could encourage further research on melatonin as a potential biomarker for the diagnosis or a possible area for the early treatment of these diseases. Many clinical studies have also produced data denoting melatonin treatment as a method to reduce the detrimental neurocognitive effects of these diseases. Further research on the role of melatonin in neurodegenerative diseases could expand symptomatic and prophylactic treatment options for diseases such as AD and PD. This review investigates melatonin's physiological properties, its role in AD and PD, and current findings on its potential therapeutic benefits in AD and PD patients.

## Introduction and background

Melatonin is a hormone produced by the pineal gland to help regulate circadian rhythm and serve as a biological signal for sleep [1]. The hormone exhibits increased levels hours before sleep and helps regulate nighttime processes through its interaction with the suprachiasmatic nucleus in the hypothalamus [2]. The relationship between melatonin and sleep may offer a new approach to studying neurodegenerative disorders, as studies have shown that sleep disorders commonly appear as early signs of neurodegenerative disease development or even as the first symptoms after diagnosis [3]. Moreover, sleep disorders have been correlated to appear in tandem with neurodegenerative diseases [4].

The utilization of exogenous melatonin has shown efficacy in treating circadian rhythm problems and improving sleep quality in patients with neurodegenerative diseases [5]. Specifically, prior studies have shown that melatonin levels in patients with Alzheimer's disease (AD) and Parkinson's disease (PD) were lower than in healthy controls, coinciding with active sleep disorders in both diseases [6,7]. Therefore, melatonin levels could be further explored in future studies as a potential biomarker in identifying disease progression and treating patients with AD and PD [6]. Current data has shown that exogenous melatonin is an efficacious and safe option to treat sleep disorders in AD and PD patients [6,7]. Aside from its role in treating sleep disorders, melatonin treatment may also exhibit an inverse relationship with pathological features of neurodegenerative diseases, such as the development of amyloid plaques [8]. While most treatments for neurodegenerative diseases are focused on symptomatic control, the role of melatonin in amyloid plaque formation offers an opportunity to further elucidate the hormone's role in mediating or modulating key mechanisms of these diseases. Therefore, this review investigates melatonin's physiological properties, its role in AD and PD, and current findings on its potential therapeutic benefits in AD and PD patients.

## Review

Melatonin: overview, pharmacokinetics, and pharmacodynamics

Melatonin Overview

**Figure 1:**
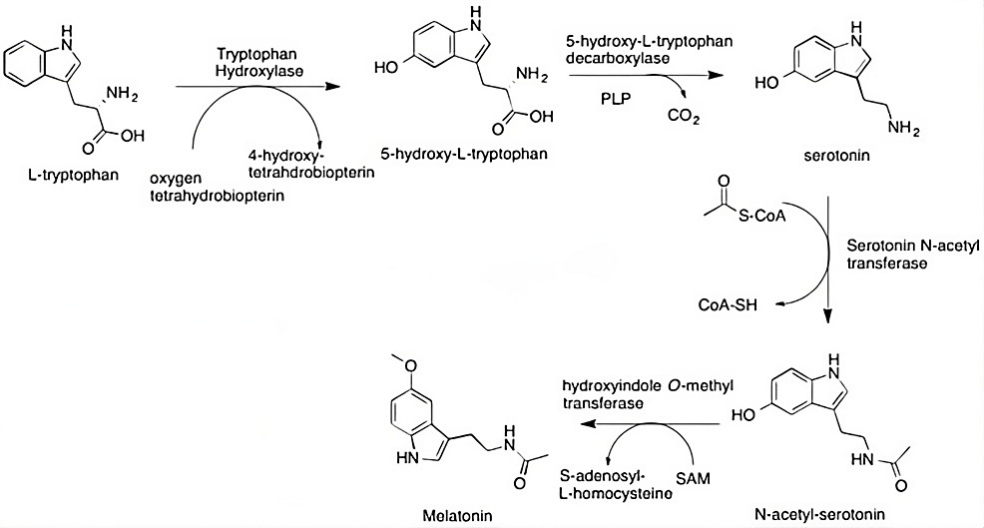
The conversion of tryptophan to melatonin is mediated by a multistep process involving the aforementioned enzymes PLP: pyridoxal phosphate; SAM: S-adenosyl methionine Image Credit: Adapted from Wikipedia as a public domain image [11]

Melatonin's production is predominantly initiated by the lack of light stimuli [9]. The formation of melatonin begins with tryptophan and proceeds with a multistep conversion process that is exhibited in Figure 1. This process is initiated upon the release of norepinephrine, which occurs at night. Norepinephrine is responsible for increasing levels of intracellular cAMP, which interacts with B-adrenergic receptors, allowing for increased cAMP-dependent protein kinase activity. This regulates the activity of the penultimate enzyme of melatonin synthesis, arylalkylamine N-acetyltransferase (AANAT). Phosphorylation of AANAT by cAMP-dependent protein kinase A allows for 14-3-3 regulatory proteins to prevent the degradation of AANAT [10].

Pharmacokinetics

Melatonin is currently considered a health supplement and thus is not regulated by the Food and Drug Administration (FDA). The dosing of oral melatonin recommended by the Cleveland Clinic begins at 1 mg and increases in 1 mg increments up to 10 mg [12]. In a study performed by Andersen et al., 12 healthy male volunteers aged 20-40 were dosed with 10 mg of oral and intravenous (IV) melatonin to evaluate the pharmacokinetic properties of this drug. The study found that the absorption half-life of 10 mg oral melatonin had a mean of six minutes, with a mean maximal concentration time of 40.8 minutes. The mean elimination half-life was 53.7 minutes, with a bioavailability of 2.5%. The nature of IV administration means that there is instant absorption. The mean elimination half-life of 10 mg IV melatonin was 39.4 minutes with a volume of distribution of 1.2 kg^-1^ [13]. The elimination of melatonin from the body is carried out by cytochrome p450 enzymes CYP1A1, CYP1A2, and CYP1B1, which work to hydroxylate melatonin to 6-hydroxymelatonin. This byproduct is then conjugated to sulfate and subsequently eliminated via urine [14].

Pharmacodynamics

The physiologic actions of melatonin can be separated into both receptor-dependent functions and receptor-independent functions. The receptor-dependent functions of melatonin include its hormonal properties, which are known to facilitate circadian rhythm [15]. There are two subtypes of this receptor, referred to as MT1 and MT2. The exact mechanism of downstream effect(s) of melatonin receptor activation is poorly understood, as the expression of melatonin-type receptors is in various tissues throughout the body. MT1 is expressed in multiple areas of the central nervous system, including the suprachiasmatic nucleus, hippocampus, cerebellum, substantia nigra, and areas within the brainstem. MT1 is also found within the retina, reproductive organs of both sexes, coronary vasculature, aorta, lungs, gallbladder, and kidneys. MT2 is found in the central nervous system and many of the same tissues listed above. Activation of MT1 results in reduced cAMP activation and increased phospholipase C activity. Activation of MT2 results in decreased cAMP production via adenylyl cyclase inhibition and modulates ion flux within cells [16]. Moreover, receptor-independent functions include melatonin's ability to serve as an antioxidant species to detoxify free radicals, which are implicated in deterioration associated with AD [15].

The role of melatonin in AD and PD

The antioxidant role of melatonin is thought to play an essential role in times of oxidative stress, including ischemic stroke, ionizing radiation, and drug toxicity [15]. Interestingly, there has been another recent study that shows decreased levels of melatonin in patients with neurologic diseases such as AD and PD [17]. With this data and other recent studies, there has been a greater focus on the absence of melatonin in times of oxidative stress in these neurodegenerative diseases.

AD

In 2022, approximately 8.5 million Americans over the age of 65 were affected by AD, and that number is expected to rise to 13.8 million by 2060 without significant advancements in treatment options [18]. AD is one of the most commonly occurring neurodegenerative diseases, marked by amyloid plaques and neurofibrillary tangles [19]. The progressiveness of this disorder primarily leads to deterioration in the medial temporal lobes, with additional damage to neocortex association areas [20].

The current medications and treatments for AD are not curative and mainly target the neurotransmitter imbalance seen with acetylcholine. The existing medications include memantine, an N-methyl-D-aspartate (NMDA) receptor antagonist, and three cholinesterase inhibitors: donepezil, rivastigmine, and galantamine [21]. The FDA has also recently approved two anti-amyloid drugs: lecanemab and aducanumab [22]. The development of new biomarkers and treatment options has led researchers to search for more identifiable markers to indicate disease onset and progression. AD pathophysiology is associated with an increased amount of oxidative stress along with other etiologies, leading to the deterioration of grey matter in the cortex. The increased stress and neuroinflammation result from the accumulation of hyperphosphorylated tau proteins and amyloid beta formation [23]. Recent data has shown that nighttime melatonin levels are decreased in AD patients compared to an age-matched healthy control patient [6,24]. These levels are reduced in the cerebrospinal fluid (CSF) of patients with even just a few neurofibrillary tangles and neuritic plaque [25]. Before even cognitive impairment begins, the melatonin levels were found to be decreased when compared to healthy controls, meaning it could serve as an early marker for AD [26]. The antioxidant benefits of naturally occurring melatonin may play a role in altering AD symptoms or disease progression, with decreased melatonin leading to increased symptoms. These effects are exhibited in the typical appearance of circadian rhythm disruptions early on in AD patients, leading to increased cognitive deterioration [8,27]. Furthermore, the accumulation of amyloid plaques is correlated to increased circadian rhythm disruptions, leading to a positive feedback loop between sleep disorder and plaque buildup [8]. Insight from this correlation could potentially lead to melatonin supplementation as a treatment either to slow disease progression or to begin treatment before AD onset.

The current state of research on the beneficial effects of melatonin has shown marked improvements in Mini-Mental State Examination (MMSE) with long-term (>12-week) treatment in patients with AD [28]. Melatonin's role in AD holds further research potential as a possible biomarker due to preclinical levels in patients showing signs of AD [25]. Moreover, melatonin might work to alleviate symptoms or even stop progression in patients due to its inverse correlation with amyloid plaque levels and its effectiveness in treating sleep disorders [8].

PD

PD is the second most common neurodegenerative disease, characterized by a resting tremor, rigidity, bradykinesia, and even postural instability in some patients [29,30]. Non-motor symptoms can be exhibited, such as PD constipation, dysosmia, cognitive impairment, and sleep disorders [31,32]. The disease process reduces the number of dopaminergic neurons, and α-synuclein accumulates within the substantia nigra pars compacta [33]. PD affects about 0.5-1.0% of those aged 65-69 and 1.0-3.0% of those aged 80 or older [29,34,35]. Similar to trends of AD discussed earlier, PD has the potential to increase in incidence and prevalence by over 30% by 2030 unless significant developments in disease prevention or treatment are found [29,36].

As seen in AD, the melatonin levels in the early stages of PD are significantly lower than melatonin levels in healthy controls [37,38]. However, later stages of PD have higher melatonin levels, which is theorized to be because of levodopa treatment [37]. These levels might coincide with the development of sleep problems before PD onset, including circadian rhythm disruptions and rapid eye movement sleep behavior disorder (RBD). Importantly, RBD is a strong α-synucleinopathy indicator, as a large proportion of RBD patients have an increased associated risk of developing PD [39]. The association between RBD and Parkinsonian pathology suggests that melatonin may have previously unappreciated therapeutic value within the discipline of neurodegenerative disease treatment. Since the appearance of sleep disorders correlates to a neurodegenerative disease onset such as PD, melatonin is being more actively studied as a therapeutic option to stop the progression of PD, with eight ongoing clinical trials to treat both PD and sleep disorders [37]. Many existing studies have shown melatonin supplementation to be efficacious in treating sleep disorders in patients with PD while maintaining a proven safety record [40,41].

Melatonin supplementation might also exhibit a role as a prophylactic treatment before serious symptoms start for PD. If these sleep disorders are caught early and linked to a precursor of PD, then melatonin supplementation could serve as a potential therapeutic to slow disease progression.

Adverse effects of chronic melatonin supplementation

When considering melatonin as a treatment in neurodegenerative diseases, potential side effects should be analyzed as well. Moderate doses of melatonin (less than 5 mg) appear safe for short periods. Some common minor side effects include headache and fatigue. However, more severe side effects have been reported, including both hypertension and hypotension, as well as dizziness. These side effects usually resolve once melatonin therapy is stopped. Chronic melatonin usage shows an increased risk of bleeding, possibly explained by a dose-dependent relationship between plasma levels of melatonin and coagulation activity [42,43]. Another study displayed that a single 3 mg dose of melatonin significantly increased disturbances in patients' ability to balance one hour after ingestion compared to placebo [44]. This side effect is particularly worrisome due to its potential impact on patients with motor dysfunction caused by neurodegenerative diseases. Other notable interactions include the potentiation of sedative effects of benzodiazepines and zolpidem [45].

Clinical studies of melatonin for AD and PD

Recent Clinical Studies on Melatonin's Role in AD

Some properties of melatonin also aid in the treatment of AD by way of its antioxidant effects and anti-inflammatory effects, both of which play a role in preventing neurodegeneration [46].

Multiple studies have investigated the different ways that melatonin has been proven to improve AD patients' sleep and cognition. A landmark case was reported by Brusco et al. on a case of monozygotic twins, both of whom had AD for eight years. The 79-year-old male twins had a difference in onset of disease by only six months; testing and evaluation indicated similar cognitive impairment and neuroimaging transformations. One of the siblings was given melatonin 6 mg orally at night for 36 months, and memory impairment was compared at the end of the three years. Both twins were assessed with the Reisberg Functional Assessment Screening Tool (FAST) for AD, and the melatonin-treated twin scored a stage 5. In contrast, the other twin scored stage 7b, indicating less disease progression in the melatonin-treated twin. In addition, both twins were given a MMSE test, of which the melatonin-treated patient scored 10/30, while the other twin scored 0/30, indicating a more severe impairment in memory without melatonin treatment [47]. Another study that evaluated cognitive improvement was reported by Sumsuzzman et al., who analyzed 22 randomized controlled trials, of which nine included AD patients, two included patients with insomnia, and 11 included healthy subjects, in the use of melatonin treatment. The AD patients who received 12+ weeks of melatonin treatment improved their MMSE scores, which indicates cognitive improvement. The AD patients had a mean age of 78.95 years. Of the different levels of AD progression, it was found that MMSE scores improved for those with mild-level AD [28]. The improvement in MMSE scores indicated melatonin's role in improving global cognitive function for mild AD. Further analysis by Blackman et al. followed the Preferred Reporting Items for Systematic Review and Meta-Analyses (PRISMA) recommendations to report on interventional studies of mild AD dementia patients being treated with different modalities, including melatonin. Melatonin treatment was investigated as a modality to optimize circadian fluctuations. Of the studies reviewed, melatonin-treated patients with mild AD illustrated reduced sleep latency and sleep-to-wakefulness transitions [48]. Sleep latency, which indicates how long it takes to fall asleep, is often impaired in patients with dementia and related diseases. Circadian rhythm disturbances are often seen in AD, as patients portray sundowning and restlessness as dementia worsens. The alteration of melatonin production in these patients can explain the abnormalities in normal sleep-wake patterns. Therefore, when patients are treated with exogenous melatonin, the circadian rhythm attempts to normalize, helping with these disturbances that accompany dementia [6]. The role of melatonin disruption at the microscopic level can also suggest further need for melatonin treatment in AD patients. Chen et al. conducted a study focused on oral melatonin treatment in the degradation of mitochondria, also known as mitophagy, in the hippocampus of AD-modeled mice versus wild type. Mitochondrial dysfunction was revealed in these experimental groups, indicating impaired mitochondria associated with AD. With oral melatonin treatment, mitophagy was repaired, and cognition improved. This study suggests the possible use of melatonin in AD in reducing the microscopic impairments that lead to cognitive deficits [49]. Melatonin has also been shown to play a role in preventing oxidative stress in the hippocampus, including hyperphosphorylation of tau proteins and amyloid beta formation, which play a role in the pathophysiology of AD [23]. Specifically, one such study by Andrade et al. reported on a streptozotocin-induced sporadic AD model in rats, which showed that melatonin treatment prevented the accumulation of amyloid plaques [50]. Table 1 below summarizes these clinical findings regarding melatonin and its implications for AD.

**Table 1:** Reported therapeutic results in melatonin treatment of AD AD: Alzheimer's disease; MMSE: Mini-Mental State Examination; PRISMA: Preferred Reporting Items for Systematic Review and Meta-Analyses

Author (year)	Groups studied and intervention	Results and findings	Conclusions
Study 1: Brusco et al. [47]	79-year-old male monozygotic twins, one of which was treated with melatonin 6 mg nightly for 36 months	Melatonin-treated twin scored lower in disease progression than the control twin (stage 5 vs 7b, respectively) as well as better in memory impairment (10/30 vs 0/30, respectively)	Melatonin plays a role in disease progression and memory impairment, as indicated in monozygotic twins with little genetic variation and a similar onset of disease
Study 2: Sumsuzzman et al. [28]	AD randomized controlled trials of melatonin treatment were analyzed according to MMSE scores	For mild-level AD patients receiving over 12 weeks of melatonin treatment, there were improvements in MMSE scores	Melatonin shows improvement in cognitive impairment scores when given to mild AD patients for over 12-week duration
Study 3: Blackman et al. [48]	Analysis following PRISMA recommendations reported on different modalities of mild AD treatment, including melatonin	Melatonin-treated patients with mild AD illustrated reduced sleep latency and less sleep-to-wakefulness transitions	Impaired melatonin production causing circadian rhythm disturbances could be improved with exogenous melatonin to reduce sleep latency and sleep-to-wakefulness transitions
Study 4: Chen et al. [49]	Oral melatonin treatment for hippocampal mitophagy in AD-modeled mice versus wild type	Melatonin-treated mice illustrated restoration of mitochondrial dysfunction and improvement in cognition	Mitochondrial dysfunction in AD was improved with melatonin treatment, suggesting a role of melatonin in treating mitophagy associated with AD
Study 5: Andrade et al. [50]	10 mg/kg melatonin infused intraperitoneally into streptozotocin-induced sporadic AD rat model	Melatonin-treated rats had reduced amyloid plaque expression in the hippocampus	Melatonin treatment successfully reduced amyloid plaque pathology and might be useful to control amyloid pathology in the brain

Recent Clinical Studies on Melatonin's Role in PD

Sleep disorders, including insomnia, daytime sleepiness, and RBD, are common in patients with PD. Similar to AD patients, there seems to be disordered melatonin secretion at night within the circadian rhythm system [51]. A randomized, double-blind, placebo-controlled trial was conducted to evaluate melatonin supplementation in PD and evaluate its effects on different aspects of the disease. The results indicated that upon 12 weeks of melatonin supplementation in patients with PD, favorable effects were indicated on the Unified Parkinson's Disease Rating Scale (UPDRS) part I score, Pittsburgh Sleep Quality Index (PSQI), Beck Depression Inventory (BDI), and Beck Anxiety Inventory (BAI). These tests evaluate patients clinically for mood, behavior, daily living, quality of sleep, depression, and anxiety. Compared to the placebo, the experimental group of patients who received melatonin treatment scored better on these testing modalities, indicating the potential benefits of melatonin supplementation. In addition to these clinical sign testing modalities, 12-week melatonin treatment resulted in reduced serum high-sensitivity C-reactive protein (hs-CRP), elevated plasma total antioxidant capacity (TAC), elevated total glutathione (GSH) levels, decreased serum insulin levels, reduced total cholesterol, and decreased low-density lipoprotein (LDL) cholesterol. Aside from clinical improvements with melatonin treatment, this study suggests improvement in serum levels with melatonin usage [52]. Additionally, a randomized controlled trial was conducted that included PD patients with a PSQI score above 5, of which the experimental group was given prolonged-release melatonin. Results were assessed, and PSQI scores significantly improved in the experimental and control groups. Additional tests conducted included the Non-Motor Symptoms Scale (NMSS) and Parkinson's Disease Quality of Life-39 (PDQ-39), both of which had significant improvements in scores for the experimental group. Prolonged-release melatonin seemed beneficial for sleep quality, non-motor symptoms, and quality of life in PD patients compared to the control group [53]. Another placebo-controlled, cross-over, randomized, double-blinded study was conducted with PD patients, in which the experimental group was given 25 mg melatonin at noon and then 25 mg melatonin 30 minutes before bedtime for three months. After a three-month treatment period, the melatonin-treated group demonstrated lower levels of lipoperoxides, nitric oxide metabolites, and carbonyl groups than the placebo group [54]. This reduction in oxidative stress markers illustrates another benefit of melatonin usage in PD patients. Mouse models were used to study the pathophysiological components of PD, including neuroinflammation. Upon melatonin treatment for seven days in mice induced with PD using 1-methyl-4-phenyl-1,2,3,6-tetrahydropyridine, there were increases in retinoid acid-associated orphan nuclear receptors, protected dopamine neurons, anti-inflammatory markers in the microglia, and a decrease in inflammation [55]. This study illustrated the neuroprotective effects that melatonin plays a role in during PD pathogenesis. Table 2 below highlights these clinical findings regarding melatonin and its role in PD.

**Table 2:** Reported therapeutic results in melatonin treatment of PD RBD: rapid eye movement sleep behavior disorder; PD: Parkinson's disease; UPDRS: Unified Parkinson's Disease Rating Scale; PSQI: Pittsburgh Sleep Quality Index; BDI: Beck Depression Inventory; BAI: Beck Anxiety Inventory; hs-CRP: high-sensitivity C-reactive protein; LDL: low-density lipoprotein; TAC: total antioxidant capacity; GSH: elevated total glutathione; NMSS: Non-Motor Symptoms Scale; PDQ-39: Parkinson's Disease Quality of Life-39

Author (year)	Groups studied and intervention	Results and findings	Conclusions
Study 1: Daneshvar Kakhaki et al. [52]	12-week randomized, double-blinded, placebo-controlled clinical trial of 60 patients with PD and RBD or restless leg syndrome either given 10 mg melatonin nightly or a placebo	Patients in the experimental group illustrated better scores on UPDRS, PSQI, BDI, and BAI as well as reduced hs-CRP, elevated TAC, elevated GSH, decreased insulin, and decreased total and LDL cholesterol	Melatonin plays a role in improving clinical scores as well as serum levels in patients with PD
Study 2: Ahn et al. [53]	PD patients with PSQI scores >5 were given prolonged-release melatonin to assess sleep scores, as well as non-motor function and quality of life	The experimental group receiving prolonged-release melatonin showed improved scores in PSQI, NMSS, and PDQ-39 as compared to the control group	Prolonged-release melatonin usage is suggestive of improving sleep, non-motor symptoms, and quality of life
Study 3: Jiménez-Delgado et al. [54]	Placebo-controlled, cross-over, randomized, double-blinded study of PD patients given 50 mg of melatonin daily for three months compared to placebo	The experimental group receiving melatonin treatment demonstrated lower levels of lipoperoxides, nitric oxide metabolites, and carbonyl groups as compared to the placebo group	Melatonin treatment has been shown to reduce oxidative stress markers in PD patients
Study 4: Li et al. [55]	PD-induced mice using 1-methyl-4-phenyl-1,2,3,6-tetrahydropyridine were treated with a seven-day course of melatonin	Treated mice illustrated increases in retinoic acid-associated orphan nuclear receptors, protected dopamine neurons, and anti-inflammatory markers as well as a decrease in inflammation	Melatonin treatment is suggested to have neuroprotective effects in PD as well as a reduction in neuroinflammatory markers

## Conclusions

Melatonin is a hormone secreted by the pineal gland predominantly in the absence of light, helping regulate the body's circadian rhythm. However, in patients with AD or PD, the level of endogenous melatonin is decreased compared to healthy controls. While this deficiency is correlated with the development of sleep disorders in neurodegenerative diseases, recent research has sought to elucidate the relationship between the appearance of sleep disorders and these diseases. Research into melatonin's role in sleep disorders has opened the door for further data collection into its potential role in the onset of neurodegenerative diseases. Furthermore, melatonin research could set a new parameter for the earlier diagnosis of these two diseases and serve as a targetable area for the prophylactic treatment of neurodegenerative diseases. Apart from melatonin's potential curative or prevention value, many clinical studies have reported its efficacy in managing symptoms of patients with either AD or PD. Moreover, the neurocognitive deterioration from sleep disorders can potentially be slowed with melatonin supplementation, which has a generally safe therapeutic profile. More research into melatonin and its role in the development of these diseases would be beneficial to uncover the extent of the therapeutic impact that melatonin supplementation can have on AD and PD patients.
